# Supplementary material for: Integrated Pest Management Strategies to Control Varroa Mites and Their Effect on Viral Loads in Honey Bee Colonies
Source: Insects. 2024 Feb 5;15(2):115. doi: 10.3390/insects15020115 (PMC10889759; doi:10.3390/insects15020115)
Supplement: Supplementary file 1 [file insects-15-00115-s001.zip › insects-2762624-supplementary.pdf]

Supplementary file S1: Protocols for virus analysis

RNA extraction protocol

Thirty adult honey bees were homogenized in 30 ml of Phosphate Buffer Solution (PBS) and 140 µl was collected for the extraction. The RNA extraction was performed with QIAamp® Viral RNA Kit (Qiagen, Hilden, Germany) and the elution was performed with 80µl of Buffer AVE.

c-DNA Synthesis protocol

Reverse transcription was carried out using a volume of 30µl of RNA with the addition of the following reagents: 6µl of 10X random hexamer primers, 6µl 10X RT-Buffer, 2,4µl dNTP mix 100mM, 3µl of 5U Multi Scribe Reverse Transcriptase and 12,6 µl of RNase-free water by High Capacity cDNA Reverse Transcription kit (Applied Biosystems™, ThermoFisher Scientific). Synthesis of yhe cDNAwas carried out using Gene Amp® PCR System 9700 (Applied Biosystems) and required the following amplification cycle: 25°C for 10 min, 37°C for 45 min and 85°C for 5 min.

Real Time ABPV protocol

The primers and probe used were designed by the Istituto Zooprofilattico delle Venezie (PD, Italy) considering the sequence NC_002548.1 relative to a non-coding region preceding the ABPVgp1gene of the Acute Paralysis Virus. The nucleotide sequences for the primers and probe were: APV-1Foward 5'-GCCCAGACAAGCGCAGTACT-3'; APV-1Reverse 5'-AGCACGGAAAACGCGTCTT-3'; ABPV-1 Probe: 5'-FAM-TCCCCGATAGCRACCGA-MGBNFQ-3'. The reaction mix for the Real Time PCR was made using the TaqMan® Universal PCR Master Mix kit (Applied Biosystems, Thermo Fisher Scientific, Foster City, CA, USA) and had the following composition: 12,5µl of TaqMan® 2X Universal PCR Master Mix, 0,9µM final concentration of 30 µM of forward primer, 0,9µM final concentration 30µM reverse primer, 0,25µM final concentration of 10 µM probe, 5 µl of cDNA or 150-200 ng of genomic DNA and H_2O G.R. DEPC up to the final volume of 25 µl. The Real Time PCR was carried out on QuantStudio™ Real Time PCR (Applied Biosystems, Thermo Fisher Scientific) and the conditions were 50°C for two minutes, 95°C for 10 minutes, and 50 cycles of 95°C for 15 seconds and 60°C for one minute. All data were analyzed using the QuantStudio™ Sequence Detection System SDS software package (Applied Biosystems, Thermo Fisher Scientific, Foster City, CA, USA). A TaqMan positive interpretation was given with a threshold cycle number (Ct) below 45 and samples were verified by examining the amplification plot options.

Real Time DWV protocol

The primers and probe used were designed by Istituto Zooprofilattico delle Venezie (PD, Italy) on the sequence NC_004830.2 relative to the DWVgp1 gene of the Deformed Wing Virus coding for a polyprotein. The nucleotide sequences for the primers and probe were: DWV Foward 5'-ATGGGTTTGATTCG/AATATCTTGGAA-3'; DWV Reverse 5'-

GATGTTCCG/AGGTGGCTTTAATGA-3'; DWV Probe 5'-FAM-ACTAGTGCTGGTTTTCCTTTGTC-NFQ-MGB. The reaction mix for the Real Time PCR was made using the TaqMan® Universal PCR Master Mix kit (Applied Biosystems, Foster City, CA, USA) and had the following composition: 12,5µl of TaqMan® 2X Universal PCR Master Mix (Applied Biosystems, Thermo Fisher Scientific, Foster City, CA, USA), 0,9 µM final concentration of 30 µM of forward primer, 0,9 µM final concentration 30 µM reverse primer, 0,25 µM final concentration of 10 µM probe, 5 µl of cDNA or 150-200 ng of genomic DNA and H2O G.R. DEPC up to the final volume of 25 µl. The Real Time PCR was carried out on the QuantStudio™ Real Time PCR (Applied Biosystems Thermo Fisher Scientific) and the conditions were 50°C for two minutes, 95°C for 10 minutes, and 50 cycles of 95°C for 15 seconds and a final step of 60°C for 1 min. All data were analyzed using the QuantStudio™ Sequence Detection System SDS software package (Applied Biosystems, Thermo Fisher Scientific, Foster City, CA, USA). A TaqMan positive interpretation was given with a threshold cycle number (Ct) below 45 and samples were verified by examining the amplification plot options.

Standard curves for ABPV and DWV

The Real Time PCRs ABPV, CBPV and DWV products were purified using the QIAquick® PCR Purification kit (Qiagen, Hilden, Germany) and were cloned in pCRII-TOPO vector (TOPO TA Cloning® Dual Promoter kit (Invitrogen, Life Technologies, Carlsbad, CA, USA). Each reactions ligation were used to transform the competent cells ONE SHOT TOP 10 (Invitrogen, Life Technologies, Carlsbad, CA, USA), following the instructions provided by the manufacturer. The presence of the PCR products of Real Time PCR ABPV and DWV were verified by sequencing of the recombinant plasmids using specific set of primers for the Real Time analyzed. The number of ABPVgp1 (ABPV) and DWVgp1 (DWV) molecules target/µl were calculated. The LOD of the Real Time PCR for ABPV, CBPV and DWV have been elaborated considering serial Log10diluitions of each of the three recombinant plasmids. In particular, the Log10 dilutions of each recombinant plasmid TOPO TA for ABPV and DWV were prepared to cover a range between 3,9 x 10^{23} to 3,9 copy/µl. In the two standard curves, there was a regression line through the data points on a plot of Ct versus the logarithm of the concentrations for each serial Log10 dilution. In detail, the Ct mean detected for ABPV and DWV corresponds to a number of target molecules/microliters in real-time PCRs. Subsequently, the amount of ABPVgp1 and DWVgp1 targets in the adult honey bee samples was determined by interpolation and corrected to target copies/30 adult honey bee samples. For ABV Table S1 reported the values standard curve.

Table S1: Values of the standard curve for ABPV.

Dilution of TOPO TA-ABPV plasmid	Ct mean	N° target molecules/µl	N. viral genomes/honey bee
tq	5.4	5.8 x 10^{23}	2.7 x 10^{24}

Dil. 10^{-1}	7.35	5.8×10^{21}	2.7×10^{22}
Dil. 10^{-2}	9.86	5.8×10^{20}	2.7×10^{21}
Dil. 10^{-3}	12.87	5.8×10^{18}	2.7×10^{19}
Dil. 10^{-4}	14.56	5.8×10^{15}	2.7×10^{16}
Dil. 10^{-5}	16.32	5.8×10^{13}	2.7×10^{14}
Dil. 10^{-6}	20.5	5.8×10^{11}	2.7×10^{12}
Dil. 10^{-7}	22.56	5.8×10^{9}	2.7×10^{10}
Dil. 10^{-8}	24.98	5.8×10^{7}	2.7×10^{8}
Dil. 10^{-9}	26.28	5.8×10^{5}	2.7×10^{6}
Dil. 10^{-10}	28.79	5.8×10^{4}	2.7×10^{5}
Dil. 10^{-11}	30.91	5.8×10^{3}	2.7×10^{4}
Dil. 10^{-12}	33.98	580	2.7×10^{3}
Dil. 10^{-13}	35.78	58	2.7×10^{2}
Dil. 10^{-14}	38.75	5.8	27

For DWV in Table S2 reported the values standard curve

Table S2: Values of the standard curve for DWV.

Dilution	Ct means	N° target molecules/μl	N. viral genomes/honeybee
Dil. 10^{-1}	4.52	$3{,}9 \times 10^{23}$	1.82×10^{24}
Dil. 10^{-2}	7.86	3.9×10^{20}	1.82×10^{21}
Dil. 10^{-3}	10.87	3.9×10^{18}	1.82×10^{19}
Dil. 10^{-4}	13.66	3.9×10^{15}	1.82×10^{16}
Dil. 10^{-5}	15.92	3.9×10^{13}	1.82×10^{14}
Dil. 10^{-6}	19.50	3.9×10^{11}	1.82×10^{11}
Dil. 10^{-7}	22.56	3.9×10^{9}	1.82×10^{10}
Dil. 10^{-8}	24.70	3.9×10^{7}	1.82×10^{8}
Dil. 10^{-9}	26.34	3.9×10^{5}	1.82×10^{6}
Dil. 10^{-10}	28.10	3.9×10^{4}	1.82×10^{5}
Dil. 10^{-11}	30.00	3.9×10^{3}	1.82×10^{4}
Dil. 10^{-12}	32.98	390	1.82×10^{3}
Dil. 10^{-13}	35.78	39	1.82×10^{2}

Dil. 10^{-14}	38.45	3.9	18.2
